# Analogue Mean Systemic Filling Pressure: a New Volume Management Approach During Percutaneous Left Ventricular Assist Device Therapy

**DOI:** 10.1007/s12265-022-10265-6

**Published:** 2022-05-11

**Authors:** Konstantin Yastrebov, Laurencie Brunel, Hugh S. Paterson, Zoe A. Williams, Chris S. Burrows, Innes K. Wise, Benjamin M. Robinson, Paul G. Bannon

**Affiliations:** 1 Department of Intensive Care, Prince of Wales Hospital, Sydney, NSW 2031 Australia; 2 The University of New South Wales, Sydney, NSW 2052 Australia; 3 Charles Perkins Research Facility, University of Sydney, Sydney, NSW 2006 Australia; 4 Royal Prince Alfred Hospital, Sydney, NSW 2050 Australia

**Keywords:** Fluid status, Analogue mean systemic filling pressure, Left ventricular assist device, Impella

## Abstract

The absence of an accepted gold standard to estimate volume status is an obstacle for optimal management of left ventricular assist devices (LVADs). The applicability of the analogue mean systemic filling pressure (Pmsa) as a surrogate of the mean circulatory pressure to estimate volume status for patients with LVADs has not been investigated. Variability of flows generated by the Impella CP, a temporary LVAD, should have no physiological impact on fluid status. This translational interventional ovine study demonstrated that Pmsa did not change with variable circulatory flows induced by a continuous flow LVAD (the average dynamic increase in Pmsa of 0.20 ± 0.95 mmHg from zero to maximal Impella flow was not significant (*p* = 0.68)), confirming applicability of the human Pmsa equation for an ovine LVAD model. The study opens new directions for future translational and human investigations of fluid management using Pmsa for patients with temporary LVADs.

**Supplementary Information:**

The online version contains supplementary material available at 10.1007/s12265-022-10265-6.

## Introduction

Intravenous fluid resuscitation represents the most frequent intervention in intensive care [1] despite known inconsistencies and the absence of a universally accepted gold standard to assess fluid status in critically ill patients, including those with mechanical circulatory support [2, 3]. Optimisation of the left ventricular preload by the administration of fluids is an important part of managing patients requiring percutaneous left ventricular assist device (LVAD) support. Hypovolemia may result in suboptimal performance of the percutaneous LVAD pump and the potential for a suctioning effect on intracardiac structures, including the mitral and subvalvular apparatus. This effect further compromises circulation and may result in iatrogenic complications [4]. Equally, excessive administration of fluids may lead to fluid overload and thus increased mortality and morbidity among critically ill adults [5].

The mean systemic pressure (Pms) is an equilibration pressure within the circulation following cardiac arrest. It is determined by the stressed blood volume and the global cardiovascular compliance. It can only be directly measured at the time of the equilibration of arterial and venous pressures within the systemic circulation following cessation of blood flow [6]. The Pms is arguably the gold standard to estimate volume status, although the method for its accurate measurement in live animals and humans has not yet been found. Several techniques have been proposed and investigated to estimate the Pms  [7]. One of the clinically applicable techniques is an analogue mean systemic filling pressure (Pmsa) calculation [8]. It has been assessed in comparative studies with other techniques estimating the Pms and has been used in clinical practice, particularly in post-cardiotomy patients [9–11] and in liver surgery patients [12]. The Pmsa appears to slightly differ in absolute values from the Pms but reliably tracks dynamic Pms changes [13].

The physiological level of the Pms in various investigations has been estimated to be 7–13 mmHg, although it has also been estimated to be 18–24 mmHg in fluid resuscitated postcardiac surgery human patients [14–17]. Continuous monitoring of the Pmsa using commercial equipment (Navigator) demonstrated highly dynamic values, constantly and rapidly changing within several mmHg. These changes are due to fluctuations in multicompartment vascular tones and are affected by vasoactive medications such as noradrenaline. Preserved right ventricular function ensures that right atrial pressure is lower than Pmsa, thus providing driving pressure for venous return which is equal to cardiac output [18]. These physiological concepts of Pmsa and venous return were recently further clinically validated [19, 20].

The Impella CP is a left ventricular assist device (LVAD), a member of the Impella family of devices. It is inserted percutaneously via a major artery (usually using the femoral or axillary arterial access), retrogradely via the aorta and aortic valve into the left ventricular cavity. The blood inlet portion of the device is positioned 3–4 cm below the aortic valve, away from the anterior mitral leaflet, while the motor and the blood outlet are in the proximal ascending aorta, connected by the 9 Fr reinforced cannula positioned across the aortic valve. The axial pump can propel up to 4.3 L/min of forward flow. This continuous forward flow can provide mechanical support for a failing left ventricle, reduce left ventricular end-diastolic volume and pressure, normalise blood flow volume for the peripheral organs, increase coronary perfusion pressure and improve physiological conditions for right ventricular function. LVADs are used for temporary mechanical support in cases of severe left ventricular failure following myocardial infarction and cardiogenic shock, to support high risk percutaneous coronary interventions, after cardiac surgery and in acute reversible myocardial insufficiency due to myocarditis and cardiomyopathy [21, 22]. They may be used in isolation or in conjunction with veno-arterial extracorporeal circulation to optimise left ventricular unloading [23]. Establishing and monitoring the causes of complex multifactorial mechanisms of haemodynamic instability in patients supported with Impella is further complicated by the absence of reliable parameter to estimate volume status.

The concept of using Pmsa as a guide to manage fluid status in patients with percutaneous LVADs, including Impella, has not been investigated. The Impella increases total cardiac output, but from a physiological aspect, this should not affect the stressed intravascular blood volume or global vascular compliance. We therefore hypothesised that various Impella flows should not result in changes to the Pms and therefore to the calculation of the Pmsa.

The aim of this study was to investigate the applicability of the human Pmsa equation in variable circulatory flows induced by a continuous flow LVAD in an ovine model in order to better inform future translational and human studies of temporary LVAD use.

## Materials and Methods

### Study Design

We conducted a prospective, interventional study in adult first-cross Merino sheep in Sydney Imaging’s Hybrid Operating Theatre at the University of Sydney, Australia. The study was approved as an extension of projects previously approved by the University of Sydney (Australia) Animal Research Ethics Committee (2019/1650 amendment) and was conducted at the Charles Perkins Centre, the University of Sydney (Sydney, Australia). The study complied with institutional general guidelines and regulations and ARRIVE guidelines for in vivo animal experiments. The study protocol and statistical analysis plan were finalised before data collection was initiated. The investigation was performed in accordance with the Helsinki Convention guidelines for humane care of animals.

### Subjects

Animals were acclimatised for at least 2 weeks prior to the procedure and received routine preventative treatments prior to arrival. Five adult female Merino first-cross sheep (1 year old, mean weight 48.6 ± 3.6 kg) underwent implantation of the Impella CP following left thoracotomy, cardiopulmonary bypass and open mitral valve surgery (mitral mechanical valve replacement and anterior mitral leaflet modifications) for a parallel investigation. Sheep were premedicated intravenously (IV) with a combination of methadone 0.2 mg/kg and midazolam 0.4 mg/kg. Anaesthesia was induced by administering propofol IV to effect, to facilitate orotracheal intubation. Anaesthesia was maintained with inspired 1–2% isoflurane delivered in an air/oxygen mixture and ketamine at an IV infusion rate of 20 µg/kg/min. Morphine 0.5 mg/kg IV was administered every 4 h. All sheep were mechanically ventilated using volume-controlled intermittent positive pressure ventilation with a positive-end-expiratory pressure of 5 cmH_2_O. Animals were anticoagulated with heparin and received fluid therapy and vasopressor support to maintain normal blood pressure and cardiac output between separate Impella runs but not between changes of Impella flows.

### Measurements

All sheep had a fluid-filled arterial pressure transducer catheter placed in the aortic arch (Teleflex 6Fr sheath introducer) and a central venous catheter placed in the left internal jugular vein (Arrow, CS-15703-E, 7 Fr 20 cm triple lumen central line. Reading, Pennsylvania USA). A transit-time ultrasonic flow probe (Transonic, Ithaca, NY) was placed around the main pulmonary artery to measure the total cardiac output. Animals were in right lateral recumbency with pressure transducers zeroed immediately before the procedure at the level of the right atrium. Haemodynamic parameters were continuously recorded. Arterial pressure, central venous pressure and cardiac output were continuously recorded (Philips Patient Monitor, IntelliVue MX800, Philips Medizin Systeme Boeblingen GmbH, Hewlett-Packard-Str.2 71,034 Boeblingen, Germany) until euthanasia as per the original research protocol.

### Impella

An Impella CP device was implanted via a 14 Fr introducer in the left common carotid artery with guidance by wide-elevation-angle three-dimensional intracardiac echocardiography [24]. Left ventricular function was stable, and there were no significant arrhythmic or other adverse event encountered during implantation. An automated Impella Controller was used to confirm the aortic pressure waveform, and colour Doppler ultrasonography was used to confirm appropriate positioning of the blood inlet and blood outlet ports.

The Impella flow was set at zero and then gradually increased in stepwise fashion using the Impella power settings, up to the maximum flow achievable (mean 3.2 L/min, range 2.7–3.6 L/min). Four stepwise increases in flow were performed in four sheep (five data points), and three stepwise increases in flow were performed in one sheep (four data points). Impella flows and corresponding haemodynamic parameters were recorded at each step following 2 min of haemodynamic stabilisation. Impella flow was then returned to zero, and the serial stepwise increases in flow were repeated. Each set of the abovementioned data points was collected over four episodes of Impella runs for each animal — a total of 96 time data points. The approach was adopted in accordance with the institutional animal ethics committee policy of maximum data acquisition using a minimum number of animals.

### Analogue Mean Systemic Filling Pressure (Pmsa)

The calculation of Pmsa has been previously described, using Eq. 1: [8]

$${\mathrm P}_{\mathrm{msa}}=(0.96\times\mathrm{CVP})+(0.04\times\mathrm{MAP})+(\mathrm c\times\mathrm{CO})$$

where CVP is the central venous pressure, MAP is the mean arterial pressure, CO is the cardiac output, and “c” is a factor to adjust the influence of resistance to venous return according to the patient’s age, height and weight.

Factor “c” in humans has been defined by the calculation in Eq. 2:

$$\mathrm c=0.038\times(94.17+0.193\times\mathrm{age})/(4.5\times\lbrack{0.9}^{(\mathrm{age}-15)}\rbrack\times0.007184\times\lbrack\mathrm{height}^{0.725}\rbrack\times\lbrack\mathrm{weight}^{0.425}\rbrack)$$

Factor “c” in human models was previously reported to be between 0.4 and 0.7 [8]. The value of factor “c” used to calculate Pmsa in this study was 0.5 for a sheep weight of 50 kg and weight-adjusted according to the following equation:

$$"c"=(0.5/50^{0.425})\times(\mathrm{weight}^{0.425})$$

Factor “c” in ovine model has been previously estimated to be 2.65 ± 1.08 [24]. The Pmsa was calculated using this value and applied to the measured data for comparison of the effects of varying factor “c”.

### The Pressure Gradient for Venous Return (VRdP)

VRdP is the difference between the mean systemic filling pressure and the right atrial pressure as expressed by the following equation:

$$\mathrm{VRdP}=\mathrm{Pmsa}-\mathrm{CVP},\mathrm{which}\;\mathrm{can}\;\mathrm{also}\;\mathrm{be}\;\mathrm{expressed}\;\mathrm{as}\;\mathrm{VRdP}=0.04(\mathrm{MAP}-\mathrm{CVP})+(\mathrm c\ast\mathrm{CO}).$$

### Systemic Vascular Resistance (SVR)

The SVR was calculated according to the following equation:

$$\mathrm{SVR}=\frac{\mathrm{MAP}-\mathrm{CVP}}{\mathrm{CO}}\times80$$

where SVR is the systemic vascular resistance, MAP is the mean arterial pressure, CVP is the central venous pressure, and CO is the cardiac output.

### Statistical Analysis

The mean values for haemodynamic variables within each sheep were calculated for each Impella CP flow increment. These mean values were used in the analyses. Normality of dependant variables was tested using the D’Agostino *K*^2^ test. Continuous variables expressed as the mean ± standard deviation or the median and interquartile range (IQR) as appropriate.

A linear regression model was fitted to examine the effect of increased Impella CP flow on each of the dependent haemodynamic variables. In these models, a term was added for each sheep to account for between-subject variation. Models were compared using the likelihood ratio test.

An *R*^2^ test was performed for a multiple linear regression model including coefficients for five sheep and a single dependent variable of interest. Assuming a model *R*^2^ of 0.50, the sample size gave 91% power to detect a significant effect on PMSA using a 5% level two-sided test.

Statistical analyses were performed using Stata 13 (College Station, Texas, USA).

## Results

Five adult 1-year-old female first-cross Merino sheep (mean body weight 48.6 ± 3.6 kg) were included with 96 complete time data points recorded for analysis at variable Impella flows. Baseline haemodynamic characteristics are presented in Table 1.

**Table 1:** Baseline haemodynamic characteristics

Variable	Value
Heart rate (bpm)	107.8 + / − 8.6
Systolic blood pressure (mm Hg)	74.0 + / − 10.3
Diastolic blood pressure (mm Hg)	42.4 + / − 3.8
Mean arterial pressure (mm Hg)	52.8 + / − 5.8
Central venous pressure (mm Hg)	9.8 + / − 1.8
Cardiac output (L/min)	4.0 + / − 1.0
Pmsa (mm Hg)	13.7 + / − 0.9

### Analogue Mean Systemic Filling Pressure (Pmsa)

**Fig. 1:**
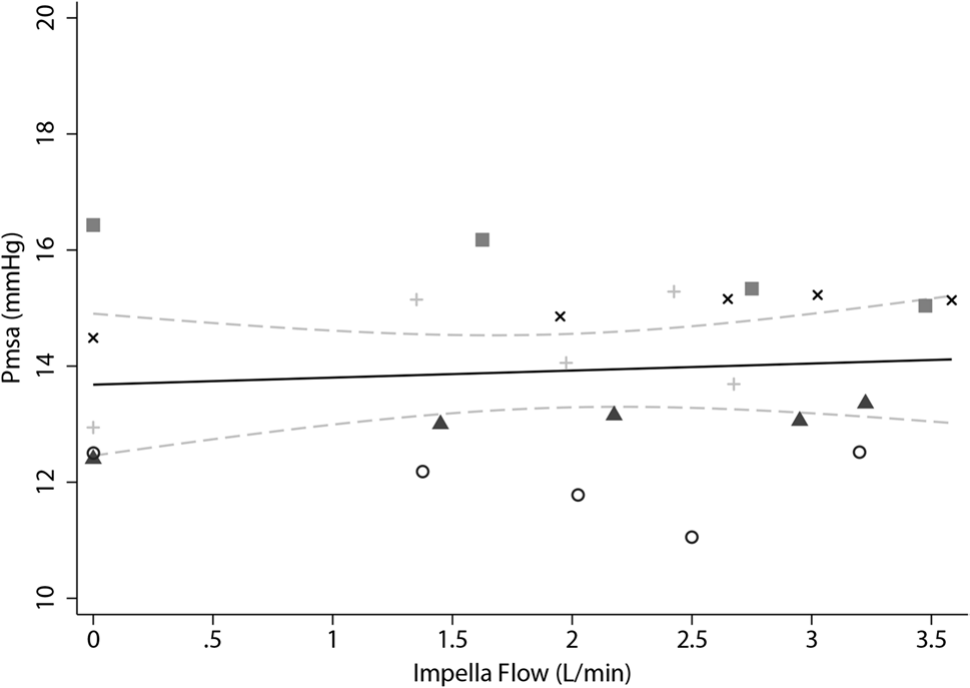
Scatterplot of mean Pmsa values and variable Impella flows with line of best fit and 95% CI. Different symbols represent individual animals. Pmsa, mean systemic filling pressure

A regression model was fitted to predict Pmsa from Impella flow (F(5,18) = 19.98, *p* < 0.0001, *R*^2^ = 0.85). The Pmsa did not change with increasing Impella flow when the Pmsa calculation used a modified equation with the anthropomorphic factor “c” adjusted for individual sheep weight [8]. The average dynamic increase in Pmsa of 0.20 ± 0.95 mmHg from zero to maximal Impella flow was not significant (*p* = 0.68) (Fig. 1). In this analysis, the mean Pmsa for all data points during the experiments was 13.9 ± 1.5 mmHg. Stepwise mean changes in the Pmsa for individual animals are summarised in Table S1 in the Supplementary Materials.

The Pmsa significantly increased with increasing Impella flow when the Pmsa calculation used a factor “c” value of 2.6 which had been estimated from a previous ovine study [21]. The mean ± SD Pmsa during all experiments was 23.45 ± 2.33 mmHg. The results were deemed physiologically incompatible with the experimental model.

### Mean Arterial Pressure (MAP)

**Fig. 2:**
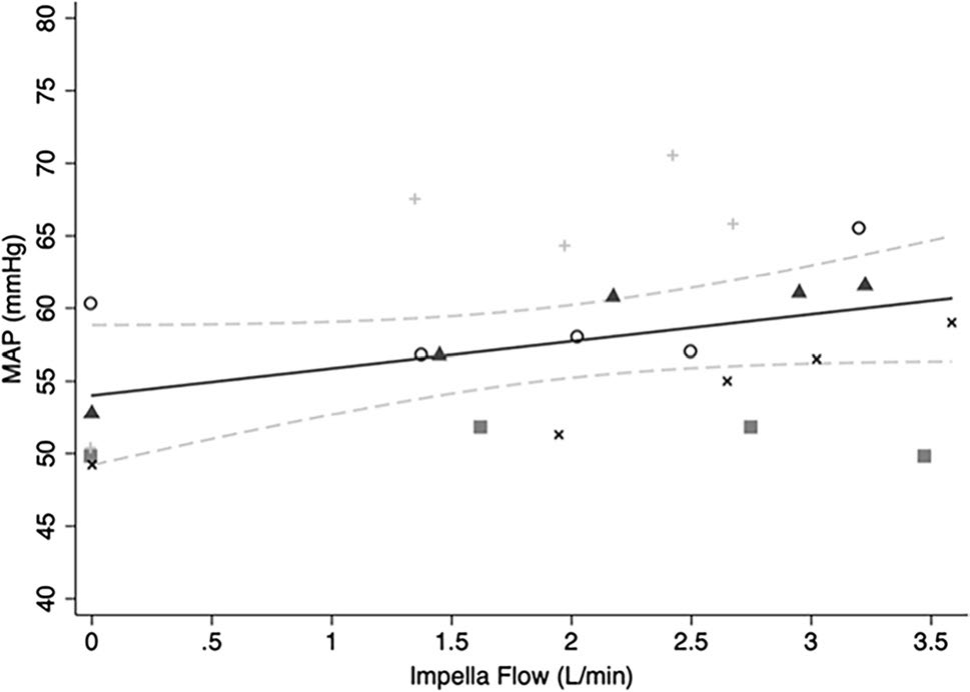
Scatterplot of mean MAP values and variable Impella flows with line of best fit and 95% CI. Different symbols represent individual animals. MAP, mean arterial pressure

The MAP significantly increased with increasing Impella flow with an average MAP increase of 7.4 ± 5.7 mmHg from zero to maximum LVAD flow. The mean changes in arterial pressures for each sheep are summarised in Table S2 in the Supplementary Materials. A regression model was fitted to examine the effect of Impella flow on MAP (F(5,18) = 13.45, *p* < 0.0002, *R*^2^ = 0.72). For each 1 L/min increase in Impella flow, MAP increased 2.36 mm Hg (95% CI 0.99–3.73, *p* = 0.002) (Fig. 2).

### Central Venous Pressure (CVP)

**Fig. 3:**
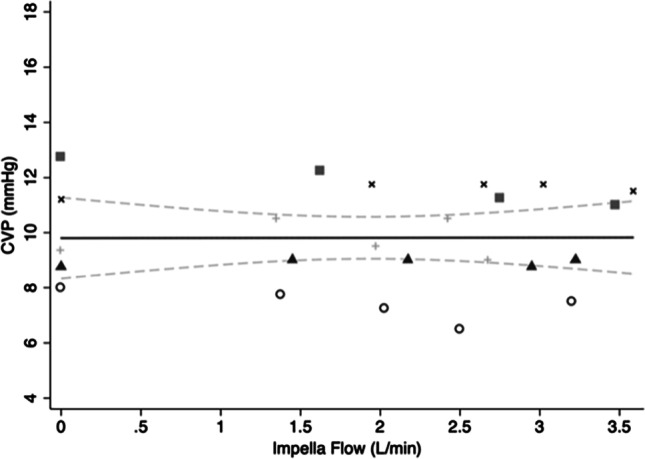
Scatterplot of mean CVP values and variable Impella flows with line of best fit and 95% CI. Different symbols represent individual animals. CVP, central venous pressure

The mean ± SD CVP decreased by − 0.41 ± 0.83 mmHg by increasing Impella flows from zero to maximum. This did not reach statistical significance on standard linear regression. The mean changes in CVP for each sheep are summarised in Table S3 in the Supplementary Materials. A regression model was fitted to examine the effect of Impella flow on CVP (F(5,18) = 52.5, *p* < 0.0001, *R*^2^ = 0.94). For each 1 L/min increase in Impella flow, CVP decreased − 0.16 mm Hg (95% CI − 0.35–0.03, *p* = 0.08) (Fig. 3).

### Total Cardiac Output (CO)

**Fig. 4:**
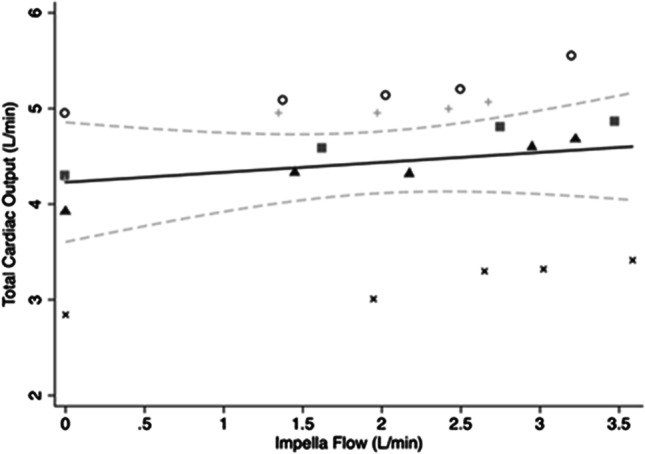
Scatterplot of total cardiac output and variable Impella flows with line of best fit and 95% CI. Different symbols represent individual animals

CO normalised for sheep weight significantly increased with increasing Impella flow. The mean ± SD total CO increased by 15.2 ± 4.2 mL/min/kg by increasing Impella flows from zero to maximum. The mean changes in CO for each sheep are summarised in Table S4 in the Supplementary Materials. A regression model was fitted to predict the effect of Impella flow on CO (F(5,18) = 206.9, *p* < 0.0001, *R*^2^ = 0.98). For each 1 L/min increase in Impella flow, CO increased 4.5 mL/min/kg (95% CI 3.4–5.6, *p* = 0.005) (Fig. 4).

### Pressure Gradient for Venous Return (VRdP)

The mean ± SD VRdP was 4.1 ± 0.6 mmHg. The mean resistance for venous return was 1.1 ± 0.07 mmHg × min × l^−1^.

**Fig. 5:**
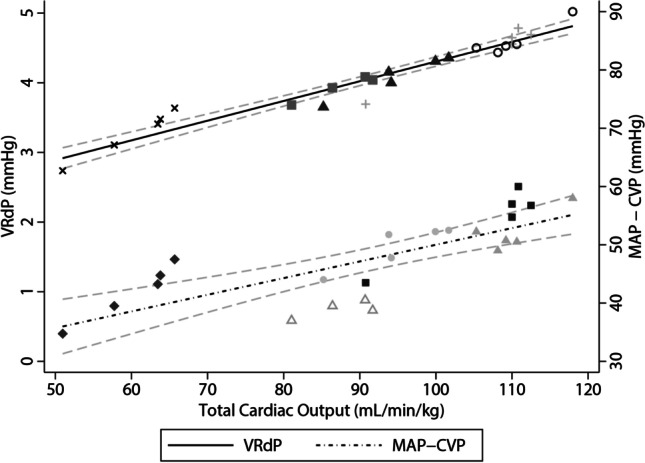
Scatterplot of gradient for venous return, total cardiac output and MAP, CVP with line of best fit and 95% CI. Different symbols represent individual animals. VRdP, pressure gradient for venous return; MAP, mean arterial pressure; CVP, central venous pressure

A regression model was fitted as for previous outcome measures to examine the effect of VRdP on CO (F(5,18) = 690, *p* < 0.0001, *R*^2^ = 0.99). There was 19.7 mL/kg/min (95% CI 17.3–22.1, *p* < 0.0001) increase in CO for each 1 mmHg increase in VRdP (Fig. 5).

A regression model was fitted as for previous outcome measures to examine the effect of MAP-CVP on CO (F(5,18) = 211, *p* < 0.0001, *R*^2^ = 0.98). There was 1.3 mL/kg/min (95% CI 1.01–1.63, *p* < 0.0001) increase in CO for each 1 mmHg increase in MAP-CVP (Fig. 5).

### Systemic Vascular Resistance (SVR)

**Fig. 6:**
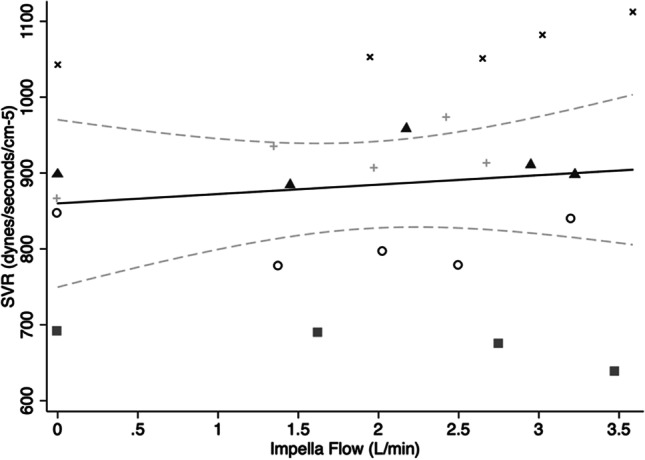
Scatterplot of systemic vascular resistance and variable Impella flows with line of best fit and 95% CI. SVR, systemic vascular resistance

The mean ± SD SVR was 884 ± 133 dynes·s·cm^−5^. A regression model was fitted as for previous outcome measures to examine the effect of Impella flow on SVR (F(5,18) = 75.11, *p* < 0.0001, *R*^2^ = 0.95). There was no significant change in SVR with alterations in Impella flow (*p* = 0.44) (Fig. 6).

### Heart Rate (HR)

The mean ± SD HR was 108.2 ± 7.3. A regression model was fitted as for previous outcome measures to examine the effect of Impella flow on HR (F(5,18) = 51.34, *p* < 0.0001, *R*^2^ = 0.93). There was no significant change in HR with alterations in Impella flow (*p* = 0.23).

The statistical summary of changes in haemodynamic variables is presented in Table S5 of the Supplementary Materials.

## Discussion

Pmsa did not change as a result of alterations in Impella flows when the Pmsa was calculated with a weight-adjusted factor “c” value similar to that previously used in human studies. This finding supports the primary study hypothesis that Pmsa is an applicable parameter for future research in subjects treated with an Impella temporary LVAD. The validation of Pmsa as a fluid status reflecting haemodynamic variable may offer clinical advancements in fluid management for patients with percutaneous LVADs to optimise functioning of the Impella and end-organ perfusion.

The validity of the finding is dependent on the use of an appropriate factor “c” value. The only previously reported estimate of an ovine factor “c” was achieved by immediate post-mortem direct measurement of Pms and reverse calculation of factor “c” based on the equation for calculation of Pmsa [25]. However, in that model, the direct measurement of Pms followed a pre-mortem period of marked cardiovascular dysfunction with extensive cardiovascular pharmacological intervention. Therefore, this “c” value of 2.6 may not have been applicable to this current model of significantly lesser-affected left ventricular and vasomotor function. When that reverse-calculated factor “c” was used to calculate Pmsa in this model, the result (23.45 mmHg) was physiologically inappropriate. Alterations in factor “c” modify the effect of the CO parameter on the calculation of Pmsa. The factor “c” used for humans usually falls within the range between 0.4 and 0.7 [8]. The mean weight-adjusted factor “c” value in this study was 0.494.

Three variables are used for the calculation of “c” in humans: age, weight and height.^8^ Estimation of an equivalent age between sheep and humans is controversial and imprecise, particularly during the first 2 years of animal life. When coupled with the differences in cardiovascular physiological implications of height between humans and animal species, a substitution of value “c” for different species in the existing equation may be invalid without other modifications to the equation.

Previous investigators used reverse calculation of “c” in dogs based on a presumed Pms of 7 mm Hg [26], while others used zero-flow technique that assumed a constant arterio-venous resistance ratio of 25.^13^ The comparison of Pmsa to zero-flow measurements of Pms in a porcine model under different loading conditions demonstrated an ability of Pmsa to track changes in the reference method with low bias but with wide limits of agreement [17]. Those experiments provided conceptual validation of Pmsa to reflect the circulatory physiology model proposed by Guyton [6]. Accordingly, our study aimed to investigate the Pmsa applicability to an LVAD model with minimal changes in loading conditions and adopted a factor “c” value consistent with that of previous researchers without direct validation by zero-flow measurements of Pms.

Two of the three haemodynamic variables used in the Pmsa equation, MAP and total CO, were found to have a statistically significant correlation with Impella performance, both variables increasing with Impella flows. The CVP decreased with increased Impella flows, as expected, but to a lesser degree and with large dispersion. CVP changes did not reach overall statistical significance when pump flow was increased from zero to maximum. Despite this, the small decrease in CVP offsets the effects of increased MAP and CO on the calculation of Pmsa. The adjusted mathematical “weight” of CVP in mmHg was approximately 4.5 times that of CO in L/min and that of MAP in mmHg. With a CVP decrease of 4.2%, a MAP increase of 15% and a CO increase of 18%, there was no significant change in Pmsa over the range of Impella flows from zero to maximum. The limited precision of measurements with standard haemodynamic monitors to reliably detect a small decrease of − 0.16 mmHg CVP for each 1 L/min increase in Impella flow could affect the accuracy of these findings.

The Impella CP is an intracardiac mechanical device intended to support the failing left ventricular intrinsic pump by providing continuous forward flow of blood into the ascending aorta from the left ventricle. The expected effects are increased total cardiac output and unloading of the left ventricular cavity throughout the cardiac cycle. CO consists of the continuous Impella flow and the remaining native systolic left ventricular ejection, and this was measured by an ultrasonic flowmeter on the main pulmonary artery. In the absence of a significant humoral response altering vascular tone, increased total forward flow should result in an increase in arterial blood pressure. With preserved right ventricular function, CVP will decrease following diastolic left ventricular unloading, leading to an increase in the gradient for venous return.

The cardiovascular response to the variable output from LVAD is complex. It involves immediate changes induced directly by the pump on flow and pressure parameters, activation of baroreceptors by pressure changes in the arterial system, the Bainbridge reflex (increased heart rate in response to increased right atrial pressure), the Anrep response (afterload inotropy) and several slow endocrine and metabolic mechanisms. There is additional complexity associated with the reduction in native pulsatile perfusion. This complexity is due to increased continuous LVAD flows and simultaneously decreased left ventricular ejection resulting from the leftward shift of Frank–Starling left ventricular contractility curve [27]. Interventricular dependence produces gradual change in the right ventricular systolic and diastolic adaptation. Resulting changes occur in the right atrial pressure which further affects baroreflex-mediated response. Improvements in the right ventricular performance due to the initiation of temporary mechanical left ventricular support by Impella have been confirmed in previous investigations [28].

Human ramp tests of LVADs showed haemodynamic changes closely corresponding with our results. Namely, a small consistent increase in cardiac output with minimal changes in CVP and an increase in diastolic and mean arterial pressure following the gradual increase in mechanical support power output and resulting LVAD flow [29]. The goal of LVAD support is usually to create a substantial reduction in left ventricular preload rather than a substantial increase in cardiac output. It is noteworthy that this reduction in preload can be associated with some increase in afterload (increase in MAP). Therefore, maintenance of the left ventricular ejection volume has complex relationship with the left ventricular work and metabolic demand.

The reduction in left atrial pressure and the progressive volume shift from the cardiopulmonary to the systemic vascular compartment are not instantaneous. We observed these changes to stabilise within an average 2 min in a pilot case prior to this investigation. Accordingly, there are compensatory mechanisms that occur within 2 min and others that occur beyond 2 min. It is problematic to measure the effects of variations in Impella flows before any compensatory mechanisms occur or to measure the effects following all compensatory mechanisms occurring. Our experiments were conducted in sheep under general anaesthesia maintained by intravenous morphine and inhaled isoflurane. Both agents are known to substantially impair baroreflexes [30, 31]. There was no significant change in HR and SVR associated with changed Impella flows after 2 min. We believe that the stability of these parameters reflects effective pharmacological blockade and thus minimised responses from baroreflexes.

A strong correlation between the VRdP and the CO with minimal bias was expected. This was based on physiological principles where CO must equate to venous return. There is mathematical coupling arising from the equation used to calculate the Pmsa. This equation contains CO as one of the variables. The equation for calculation of VRdP (VRdP = Pmsa − CVP) can also be expressed as VRdP = 0.04(MAP − CVP) + (c*CO). The independent contribution of (MAP – CVP) to the equation is shown in Fig. 5.

It was also informative to find that each 1 mmHg increase in VRdP resulted in an estimated 0.96 L/min increase in CO in our model. Thus, the VRdP may present an interesting haemodynamic target during volume and vasopressor resuscitation when aiming for a specific alteration in CO. However, the caveats of dynamic changes in resistance to venous return and shifts in right ventricular position on the Frank–Starling curve must be considered. These aspects could influence this relationship between VRdP and CO, including for patients with LVADs. There have been no prior studies of Pmsa in association with Impella LVAD support, and we were reluctant to add complexity to this study by investigating the performance of Pmsa in these subjects under different loading conditions, which should be the aim of future studies.

The strengths of our study include a pre-specified protocol and statistical analysis plan. The study was conducted over the short inception period with high levels of data integrity. Animals had similar baseline characteristics which allowed adequate statistical power to address the primary hypothesis that is applicable for an explorative analysis. Confounding bias was mitigated by standardising operative and investigative techniques using expert cardiac surgeons, echocardiographers and veterinary anaesthetists to perform previously refined procedures.

The limitations of the study include a small number of experimental animals as dictated by the principles used by the institutional animal ethics committee. The application of a mixed-effect model in the limited number of animals where multiple measurements were made in serial experiments was essential for best statistical integrity of the results. There is no known and validated approach to correctly estimate individual factor “c”, required for Pmsa calculations in an ovine model. It prompted the use of an average “c” value previously estimated for humans and normalised for the weight of the animals. This provided an appropriate estimate of Pmsa from the baseline haemodynamic parameters. The precision of direct CVP measurements was limited by the standard haemodynamic monitoring and by the complexity of minor changes in the CVP waveform during each cardiac cycle. Direct measurement of Pms at zero-flow was not performed in this study. These measurements would have validated or modified the use of factor “c” in this study and would have strengthened the significance of the results. Indeed, the main findings largely depend on the validity of the measurements of factor “c” by other researchers and on the applicability to this model.

## Conclusion

The study confirmed applicability of human Pmsa equation in an ovine left ventricular assist device model, justifying further investigation of Pmsa as a haemodynamic variable reflecting fluid status — to optimise Impella performance and end-organ perfusion during translational and human clinical percutaneous LVAD therapy.

## Supplementary Information

Below is the link to the electronic supplementary material.

Supplementary file1 (DOCX 20 KB)

## Data Availability

The authors will provide a complete set of raw data on reasonable request.
