# Supplementary material for: Analogue Mean Systemic Filling Pressure: a New Volume Management Approach During Percutaneous Left Ventricular Assist Device Therapy
Source: J Cardiovasc Transl Res. 2022 May 11;15(6):1455–63. doi: 10.1007/s12265-022-10265-6 (PMC9722875; doi:10.1007/s12265-022-10265-6)
Supplement: Supplementary file 1 — Supplementary file1 (DOCX 20 KB) [file 12265_2022_10265_MOESM1_ESM.docx]

**Supplementary material:**

**Table S1. Individual sheep dynamic changes in analogue mean systemic filling pressure:**

| **Sheep number** | **The Mean ± SD Pmsa at zero Impella flow (mmHg)** | **The Mean ± SD Pmsa at step 1 Impella flow (mmHg)** | **The Mean ± SD Pmsa at step 2 Impella flow (mmHg)** | **The Mean ± SD Pmsa at step 3 Impella flow (mmHg)** | **The Mean ± SD Pmsa at maximum Impella flow (mmHg)** | **The Mean ± SD (mmHg)** | **Pmsa change with Impella flow increase from zero to a maximum (mmHg)** |
| --- | --- | --- | --- | --- | --- | --- | --- |
| **1** | 16.4 ± 1.0 | 16.2 ± 0.4 | 15.3± 0.5 |  | 15.0 ± 0.2 | 15.7 ± 0.8 | -1.4 |
| **2** | 12.4 ± 0.6 | 13.0 ± 0.2 | 13.2 ± 0.3 | 13.1 ± 0.7 | 13.4 ± 0.3 | 13.0 ± 0.5 | 0.6 |
| **3** | 14.5 ± 1.1 | 14.9 ± 1.3 | 15.2 ± 1.5 | 15.2 ± 1.4 | 15.1 ± 0.8 | 15.0 ± 1.1 | 0.6 |
| **4** | 12.9 ± 1.0 | 15.1 ± 2.1 | 14.1 ± 2.0 | 15.3 ± 2.6 | 13.7 ± 1.1 | 14.2 ± 1.9 | 1.3 |
| **5** | 12.5 ± 0.9 | 12.1 ± 1.1 | 11.8 ± 1.3 | 11.1± 1.5 | 12.5 ± 1.3 | 12.0 ± 1.2 | 0 |

*Definition of abbreviations: Pmsa = analogue mean systemic filling pressure*

**Table S2. Individual sheep dynamic changes in mean arterial pressure:**

| **Sheep number** | **MAP at zero Impella flow**  **(mmHg)** | **MAP at maximum Impella flow**  **(mmHg)** | **MAP difference following change in Impella flow (mmHg)** |
| --- | --- | --- | --- |
| **1** | 49.8 ± 4.1 | 49.9 ± 2.6 | 0 |
| **2** | 52.8 ± 3.8 | 61.5 ± 5.7 | + 8.7 |
| **3** | 46.5 ± 4.9 | 59 ± 2.9 | + 12.5 |
| **4** | 52.8 ± 5.9 | 65.8 ± 5.9 | + 18 |
| **5** | 60.3 ± 1.7 | 65.5 ± 6.5 | + 5.3 |

*Definition of abbreviations: MAP = mean arterial pressure*

**Table S3. Individual sheep dynamic changes in central venous pressure:**

| **Sheep number** | **CVP at zero Impella flow**  **(mmHg)** | **CVP at maximum Impella flow**  **(mmHg)** | **CVP difference following change in Impella flow (mmHg)** |
| --- | --- | --- | --- |
| **1** | 12.8 ± 1.09 | 11 ± 0 | - 1.8 |
| **2** | 8.8 ± 0.5 | 9 ± 0 | + 0.2 |
| **3** | 11.8 ± 1.5 | 11.5 ± 1.0 | - 0.3 |
| **4** | 9.3 ± 1.0 | 9 ± 0.8 | - 0.3 |
| **5** | 8 ± 0.8 | 7.5 ± 1.0 | - 0.5 |

*Definition of abbreviations: CVP = central venous pressure*

**Table S4. Individual sheep dynamic changes in normalised total cardiac output:**

| **Sheep number** | **Total CO at zero Impella flow**  **(mL/kg/min)** | **Total CO at maximum Impella flow**  **(mL/kg/min)** | **Total CO difference following change in Impella flow from zero to maximum (mL/kg/min)** |
| --- | --- | --- | --- |
| **1** | 81.0 ± 7.2 | 91.7 ± 3.1 | + 10.7 |
| **2** | 85.2 ± 2.3 | 101.7 ± 5.7 | + 16.5 |
| **3** | 50.1 ± 5.9 | 65.7 ± 3.9 | + 15.6 |
| **4** | 90.8 ± 0.4 | 112.5 ± 14.6 | + 21.7 |
| **5** | 105.3 ± 5.1 | 118.0 ± 11.1 | + 12.7 |

*Definition of abbreviations: CO = cardiac output, mL/min/kg = millilitres per kilogram per minute*

**Table S5. Statistical summary of changes in haemodynamic variables following increased Impella flow from zero to maximum.**

| **Variable** | **Value** | **Absolute**  **change** | **%** | ***p* value** | **Regression model** | | |
| --- | --- | --- | --- | --- | --- | --- | --- |
|  |  |  |  |  | **Correlation** | ***p* value** | **R2** |
| **Heart rate (bpm)** | 108.2 ± 7.3 | -1.5 ± 4.8 | -1.4 | 0.226 | F(5,18)=51.34 | <0.0001 | 0.93 |
| **Systolic blood pressure (mm Hg)** | 74.4 ± 10.0 | -0.1 ± 7.2 | <1 | 0.912 | F(5,18)=22.37 | <0.0001 | 0.86 |
| **Diastolic blood pressure (mm Hg)** | 42.4 ± 3.3 | 12.4 ± 4.8 | 29.3 | <0.0001 | F(5,18)=15.24 | <0.0001 | 0.81 |
| **Mean arterial pressure (mm Hg)** | 52.4 ± 5.1 | 7.4 ± 5.7 | 15.1 | 0.001 | F(5,18)=13.45 | <0.0001 | 0.72 |
| **Central venous pressure (mm Hg)** | 9.7 ± 2.0 | -0.41 ±0.83 | -4.2 | 0.088 | F(5,18)=52.5 | <0.0001 | 0.94 |
| **Cardiac output (L/min)** | 4.0 ± 0.8 | 0.7 ± 2 | 17.5 | <0.0001 | F(5,18)=206.9 | <0.0001 | 0.98 |
| **Pmsa (mmHg)** | 13.8 ± 1.7 | 0.2 ± 0.95 | 1.4 | 0.678 | F(5,18)=19.98 | <0.0001 | 0.85 |
| **SVR (dynes/seconds/cm^-5^)** | 884 ± 133 | 11 ± 48 | 1.3 | 0.444 | F(5,18)=75.11 | <0.0001 | 0.95 |
| **VRdP (mmHg)** | 4.1 ± 0.6 | 0.7 ± 0.3 | 18.9 | <0.0001 | F(5,18)=690 | <0.0001 | 0.99 |

*Definition of abbreviations: Pmsa – analogue mean systemic filling pressure, SVR – systemic vascular resistance, VRdP – pressure gradient for venous return*
